# An In Vitro Analysis of Surface Treatment Effects on the Tensile Bond Strength Between Heat-Cured Denture Base and Heat-Polymerized Polymethyl Methacrylate Liner

**DOI:** 10.7759/cureus.98898

**Published:** 2025-12-10

**Authors:** Sumit Verma, Manmeet Gulati, Manmohit Kumar, Ram R Goyal, Aalok Mishra, Silky Grover, Manish Sharma

**Affiliations:** 1 Department of Prosthodontics, Desh Bhagat Dental College and Hospital, Mandi Gobindgarh, IND; 2 Department of Oral Pathology, Jawahar Medical Foundation's Annasaheb Chudaman Patil Dental College, Dhule, IND

**Keywords:** liner, polymethyl methacrylate, resin, surface, treatment

## Abstract

Introduction: Successful relining of complete dentures requires strong interfacial adhesion between the existing heat-cured polymethyl methacrylate (PMMA) denture base and the newly applied heat-polymerized PMMA liner to prevent delamination under functional stress. This in vitro investigation examined the efficacy of different surface conditioning methods in enhancing bond integrity and microstructural characteristics at the resin-resin interface.

Materials and methods: 48 rectangular specimens were prepared from heat-cured PMMA resin and subjected to standardized long-cycle polymerization. Each specimen was sectioned at the midline to expose a uniform bonding surface and randomly allocated to six experimental groups (n = 8): no treatment (control), methyl methacrylate monomer application, 36% phosphoric acid etching, 50 µm alumina particle sandblasting, erbium, chromium, yttrium-scandium-gallium-garnet (Er,Cr:YSGG) laser irradiation followed by phosphoric acid etching, and laser irradiation followed by alumina sandblasting. All procedures used calibrated equipment, digital timing, and custom positioning jigs to ensure reproducibility. The liner was applied using an identical resin from the same batch, trial-packed under controlled pressure, and polymerized using the same technique. The specimens were stored in distilled water for 30 days before testing. The tensile bond strength was determined by three-point bending using a universal testing machine. The selected samples underwent gold sputter coating and examination using field-emission scanning electron microscopy.

Results: The tensile bond strength varied significantly across groups (p < 0.001). The untreated control recorded the lowest mean tensile strength at 0.92 ± 0.06 megapascals (MPa). Monomer application achieved the highest value of 1.89 ± 0.04 MPa, followed by laser plus phosphoric acid at 1.74 ± 0.03 MPa, laser plus sandblasting at 1.41 ± 0.04 MPa, sandblasting alone at 1.24 ± 0.03 MPa, and phosphoric acid alone at 1.15 ± 0.02 MPa. Post-hoc comparisons confirmed the statistical superiority of the monomer group over all other modalities, and positioned the laser-phosphoric acid combination as the next most effective approach. Scanning electron microscopy revealed smooth, non-retentive control surfaces with adhesive failure, whereas monomer-treated interfaces displayed polymer interdiffusion and cohesive failure. Laser-phosphoric acid samples exhibited deep micro-undercuts with extensive resin penetration, whereas sandblasting produced superficial grooves with incomplete infiltration.

Conclusion: Monomer application was found to be the most reliable and clinically practical method for maximizing liner adhesion through chemical interpenetration. The laser phosphoric acid protocol provides a viable alternative when advanced equipment is available, combining precise ablation with enhanced wettability.

## Introduction

The longevity and clinical success of complete dentures largely depend on the integrity of the denture base, which is prone to fractures under masticatory loads. Heat-cured polymethyl methacrylate (PMMA) remains the gold standard for denture bases owing to its excellent aesthetics, ease of processing, and biocompatibility [1]. Despite these advantages, clinical challenges, such as dimensional changes, residual stress, and patient-related factors, often necessitate the application of a heat-polymerized denture base liner to reline or modify existing prostheses. Successful integration of the liner with the original heat-cured base requires robust interfacial adhesion to withstand occlusal forces and prevent delamination during function [2]. The bond strength at this interface is influenced by the surface characteristics of the existing PMMA, including topography, chemical reactivity, and wettability [3].

Surface treatments are employed to enhance micromechanical retention and chemical bonding by altering the substrate [2,3]. Phosphoric acid etching selectively dissolves the polymer surface, creating micropores that facilitate resin infiltration [4]. Sandblasting with alumina particles mechanically roughens the surface, increases the surface area, and promotes interlocking [5]. According to previous systematic reviews, sandblasting does not lead to a significant increase in the bond strength of the soft liner to the denture base resin [5], whereas the application of methyl methacrylate monomer softens and swells the PMMA matrix, enabling diffusion and chain entanglement with the liner resin, thereby increasing the bond strength between the denture base resin and the liner [3]. In contrast, Nakhaei et al. reported a higher tensile strength when the surface of the denture base resin was altered using air abrasion [6]. Laser surface treatment has emerged as a modern alternative, utilizing controlled energy to ablate and modify the PMMA surface, creating microretentive patterns without introducing abrasive particles [3].

Combining chemical etching with mechanical abrasion or laser ablation may yield synergistic effects by exposing fresh reactive sites while maximizing roughness. Scanning electron microscopy (SEM) is a valuable tool for visualizing treatment-induced morphological changes and evaluating interfacial adaptation post-bonding, providing insights into failure mechanisms [7]. Although individual treatments have been investigated, comparative studies specifically addressing heat-polymerized denture base liners bonded to heat-cured PMMA substrates with integrated SEM analysis are limited. Standardized evaluation of these modalities is essential to establish evidence-based protocols for predictable linear adhesion in prosthodontic practice.

The aim of this study was to evaluate the effects of phosphoric acid etching, sandblasting with alumina particles, monomer application, laser irradiation, and combinations of laser and phosphoric acid or alumina sandblasting on the tensile strength of heat-cured PMMA denture base resin and heat-polymerized denture base liner, supplemented by SEM analysis of surface and interfacial morphology. The objectives were to measure and compare tensile strengths across treatment groups, characterize pre- and post-treatment surface topography and roughness using SEM, correlate microstructural observations with mechanical performance, and recommend the optimal surface preparation technique for clinical liner application. The null hypothesis stated that the various surface treatment techniques would not produce significant differences in tensile bond strength or interfacial morphology at the PMMA-liner interface.

## Materials and methods

This in vitro experimental study was conducted in the Department of Prosthodontics at Desh Bhagat Dental College and Hospital, Mandi Gobindgarh, Punjab, India, over six months in 2024. As the study did not involve any human participants, ethical approval was not required for the study.

Sample size estimation

The sample size was estimated using the Gpower software (version 3.1.9.2; Heinrich Heine University, Düsseldorf, Germany). A priori analysis of the F test family, fixed effect omnibus for six groups, estimated a minimum sample size of 48 (8 per group). An effect size of 0.55 was obtained from the reference study by Almuraikhi T [8]. The tensile strength of the soft liner was analyzed after treatment with three different surface protocols (alumina, phosphoric acid, and the control group). The present study, at 80% power and 95% confidence, provided a sample size of 48 using the following formula:

\begin{equation} n = \frac{(Z_{1-\beta} + Z_{1-\alpha/2})^2 \cdot (k-1) \cdot \sigma^2}{k \cdot \delta^2} \end{equation}

where n = sample size, k = number of groups, Z₁₋α/₂ = confidence interval at 95% (1.96), Z₁₋β = power of study at 80% (0.84), σ = pooled standard deviation, and 𝛿 = mean difference.

**Figure 1:**
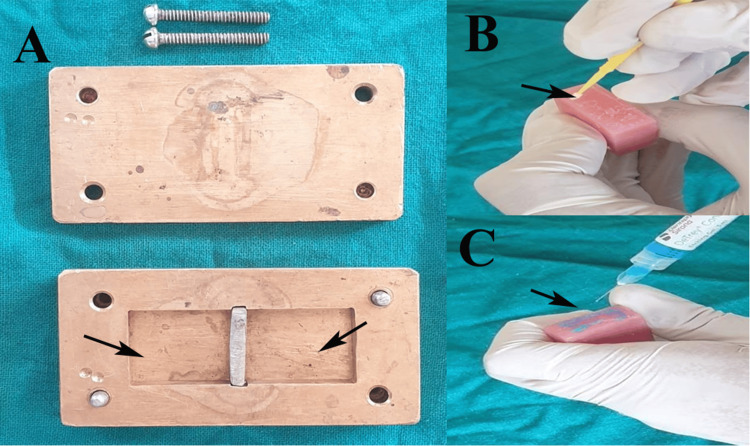
Procedure for fabrication of samples and surface treatment. (A) Metal mould for fabrication of acrylic blocks, (B) application of monomer on surface of acrylic block, (C) application of phosphoric acid on surface of acrylic block. Original images of samples from the study.

Forty-eight rectangular specimens (65 mm × 10 mm × 3.3 mm per ISO 20795-1) using heat-cured PMMA denture base resin (DPI Heat Cure; The Bombay Burmah Trading Corp. Ltd., Mumbai, India) were fabricated in a metal mold (Figure 1A). The powder and liquid were mixed in a 3.5:1 ratio by volume and packed into brass molds under a hydraulic pressure of 100 bar. The specimens underwent long-cycle polymerization in an acrylizer (Unident Instruments India Pvt. Ltd., New Delhi, India) at 74^o^C for 8 h and then at 100^o^C for 1 h, followed by bench cooling to avoid warping. The finishing of samples was done with 400- then 600-grit silicon carbide paper under water and polished with pumice slurry for a uniform 3 mm ± 0.1 mm thickness (measured with a digital vernier caliper, Mitutoyo Corp., Kawasaki, Japan; accuracy ±0.02 mm). Each specimen was sectioned transversely in the midline with a diamond disc, creating a 10 mm × 10 mm bonding surface with a 0.5 mm butt-joint gap, and 2 mm diameter holes at the ends were drilled for universal testing machine (UTM) gripping.

The specimens were randomly divided into five groups (n = 8 each) using a computer-generated table: Group 1 (Control), where no surface treatment was performed on acrylic block surface (10 mm x10 mm area); Group 2 (Monomer), where methyl methacrylate monomer (DPI Monomer; The Bombay Burmah Trading Corp. Ltd., Mumbai, India) was applied with a microbrush for 180 s until glossy (Figure 1B), then air-dried for 30 s; Group 3 (phosphoric acid), where samples were etched with 36% phosphoric acid (DeTrey Conditioner; Dentsply Sirona, Charlotte, NC, USA) for 30 s, rinsed 15 s, and air-dried (Figure 1C); and Group 4 (Sandblasting), where samples were blasted with 50 μm alumina particles (Korox 50; BEGO GmbH & Co. KG, Bremen, Germany) at 0.4 MPa, 10 mm distance, perpendicular for 10 seconds, then air-cleaned,, and Group 5 (Combination), where samples were irradiated with erbium, chromium:yttrium-scandium-gallium-garnet (Er,Cr:YSGG) laser (Biolase iPlus; 2780 nm, 3.0 W, 30 Hz, 100 μs pulse, 60% air, 40% water, non-contact, MZ6 tip, 10 mm distance, scanned in overlapping horizontal and vertical patterns for 20 seconds), followed by phosphoric acid etching as in Group 3, and Group 6, which received laser irradiation (same parameters), followed immediately by sandblasting as in Group 4. All treatment applications were standardized using digital timers (accuracy ±0.1 s), custom acrylic jigs for distance and angle control, and a motorized scanning stage for uniform laser delivery. The same batch of DPI heat-cure material was used for both the base and liner to eliminate batch variability. The motorized scanning stage was calibrated to a linear travel speed of 10 mm/s, enabling consistent, uniform laser irradiation across all specimens.

For the liner application, the treated halves were repositioned in the mold to maintain alignment. Fresh heat-cured PMMA (same DPI heat-cure batch) was applied to the bonding area in the dough stage, trial-packed twice at 100 bar, and polymerized using an identical cycle. A hydraulic molding press capable of delivering 100 bar pressure was used during both trial packings. This pressure is routinely recommended for heat-cured PMMA processing to eliminate voids, ensure complete adaptation, and achieve a uniform and intimate interface before polymerization. The finished specimens were stored in distilled water at 37^o^C for 30 days before testing to simulate oral conditions. The tensile bond strength was tested using three-point bending on a UTM (Paramount Digi-Strength; Paramount Industries, New Delhi, India) at a crosshead speed of 5 mm/min and a span of 50 mm. The peak load at fracture (N) was recorded, and the strength was calculated in megapascals (MPa). For SEM analysis, two additional specimens per group were prepared. Gold sputtering was performed on samples (10 nm coating) and imaged with an SEM (JSM-7610FPlus; JEOL Ltd., Tokyo, Japan) at 15 kV, 2000X magnification for pre-treatment surfaces and fractured interfaces, ensuring identical working distance (10 mm) and beam current calibration. A single operator performed all the procedures under controlled conditions (23 ± 2^o^C, 50 ± 10% relative humidity) to ensure reproducibility. All equipment was calibrated before testing: an acrylizer with a digital thermometer (±0.5^o^C accuracy), sandblasting unit with a pressure gauge (0.4 MPa ± 0.05), laser unit with a power meter (Nova II; Ophir Optronics Solutions Ltd., Jerusalem, Israel) to verify output stability (±0.1 W), and UTM load cell per manufacturer protocols with verification loads for zero error and linearity.

Statistical analysis

Data were analyzed using IBM SPSS Statistics for Windows, version 26 (IBM Corp., Armonk, NY, USA). The results are expressed as the mean ± standard deviation along with the corresponding confidence intervals. Data normality was assessed using the Shapiro-Wilk test and confirmed to follow a normal distribution. A parametric test, one-way analysis of variance (ANOVA), was applied to compare the tensile strength across the groups, while intragroup pairwise comparisons were performed using post-hoc Tukey’s test. Statistical significance was set at p < 0.05, and multiple comparisons were adjusted to ensure robustness of the findings.

## Results

Descriptive analysis of the tensile strength revealed significant variations based on the surface treatment applied to the heat-polymerizing acrylic soft denture liner. The control group exhibited the lowest mean tensile strength (0.92 ± 0.06 MPa). Among the chemical treatments, monomer application resulted in the highest mean tensile strength (1.89 ± 0.04 MPa), substantially outperforming phosphoric acid (1.15 ± 0.02 MPa) and sandblasting (1.24 ± 0.03 MPa). For the combined laser and chemical treatments, the laser with phosphoric acid yielded a higher mean tensile strength (1.74 ± 0.03 MPa) than the laser with alumina (1.41 ± 0.04 MPa). The inference is that the surface treatments profoundly enhanced the tensile strength of the material compared to the untreated control. The monomer treatment was the most effective single process, whereas the combination of laser with phosphoric acid was the most effective hybrid treatment, although it still did not surpass the monomer group. This suggests that chemical bonding of the monomer provides a superior adhesive interface for the liner (Table 1).

**Table 1:** Descriptive statistics of tensile bond strength in megapascals (MPa) between heat-cured polymethyl methacrylate denture base and heat-polymerized polymethyl methacrylate liner under different surface treatments. The tensile strength has been presented as mean and standard deviation (SD), number of samples in each group has been presented as frequency (n) and percentage (%), CI: confidence interval.

Group (surface treatment)	Samples per group as n (%)	95% CI for mean	Mean ± SD
Control	8 (16.66%)	0.87 - 0.97	0.92 ± 0.06
Monomer	8 (16.66%)	1.85 - 1.93	1.89 ± 0.04
Phosphoric acid	8 (16.66%)	1.13 - 1.17	1.15 ± 0.02
Sandblasting	8 (16.66%)	1.21 - 1.26	1.24 ± 0.03
Laser and phosphoric acid	8 (16.66%)	1.71 - 1.77	1.74 ± 0.03
Laser and sandblasting	8 (16.66%)	1.38 - 1.44	1.41 ± 0.04

One-way ANOVA results indicated a statistically significant difference in tensile strength between the surface treatment groups (p = 0.001). The extremely high eta-squared value (η² = 0.99) implies that the type of surface treatment accounted for nearly all of the variance observed in the tensile strength measurements (Table 2). Therefore, the null hypothesis is rejected.

**Table 2:** One-way analysis of variance (ANOVA) comparing tensile bond strength across surface treatment groups. *p < 0.05 denotes statistically significant results using one-way ANOVA, df: degree of freedom, η² = eta-squared (effect size), where η² = 0.99 indicates surface treatment explains 99% of variance in bond strength.

Variables	Sum of squares	df	Mean square	F stats	p-value	η^2^
Group	5.39	5	1.08	719.19	0.001*	0.99
Residual	0.06	42	0			

Based on post-hoc analysis, all pairwise comparisons between the six surface treatment groups demonstrated a statistically significant difference in the mean tensile strength (p = 0.001 for all pairs). The monomer treatment group was a clear outlier, exhibiting a significantly higher tensile strength than the other groups, as evidenced by its substantial positive mean differences. The combined laser and phosphoric acid treatment was the second most effective, showing a significantly higher mean tensile strength than all groups except the monomer, against which it had a smaller, yet still significant, difference (0.15 MPa). The control group consistently demonstrated the lowest tensile strength, with all comparisons showing significant negative differences. Although all surface treatments significantly improved the tensile strength compared to the untreated control, their efficacy varied considerably. Monomer application was the most effective single treatment, and its performance was statistically superior to that of the best combined laser-chemical treatment (Table 3).

**Table 3:** Post-hoc Tukey’s test for pairwise comparisons of transverse bond strength between surface treatment groups. *p < 0.05 denotes statistically significant results using post-hoc analysis by Tukey’s test with Bonferroni-adjusted p-values, CI: confidence interval, positive mean difference denotes higher bond strength in the first group.

Pairwise groups	Mean difference (MPa)	p-value	95% CI lower limit	95% CI upper limit
Control - monomer	0.97	0.001*	0.91	1.03
Control - phosphoric acid	0.23	0.001*	0.17	0.29
Control - sandblasting	0.32	0.001*	0.26	0.38
Control - laser and phosphoric acid	0.82	0.001*	0.76	0.88
Control - laser and sandblasting	0.49	0.001*	0.43	0.55
Monomer - phosphoric acid	0.74	0.001*	0.68	0.80
Monomer - sandblasting	0.65	0.001*	0.59	0.71
Monomer - laser and phosphoric acid	0.15	0.001*	0.09	0.21
Monomer - laser and sandblasting	0.48	0.001*	0.42	0.54
Phosphoric acid - sandblasting	0.09	0.001*	0.03	0.14
Phosphoric acid - laser and phosphoric acid	0.59	0.001*	0.53	0.65
Phosphoric acid - laser and sandblasting	0.26	0.001*	0.20	0.32
Sandblasting - laser and phosphoric acid	0.50	0.001*	0.44	0.56
Sandblasting - laser and sandblasting	0.17	0.001*	0.11	0.23
Laser and phosphoric acid - laser and sandblasting	0.33	0.001*	0.27	0.39

**Figure 2:**
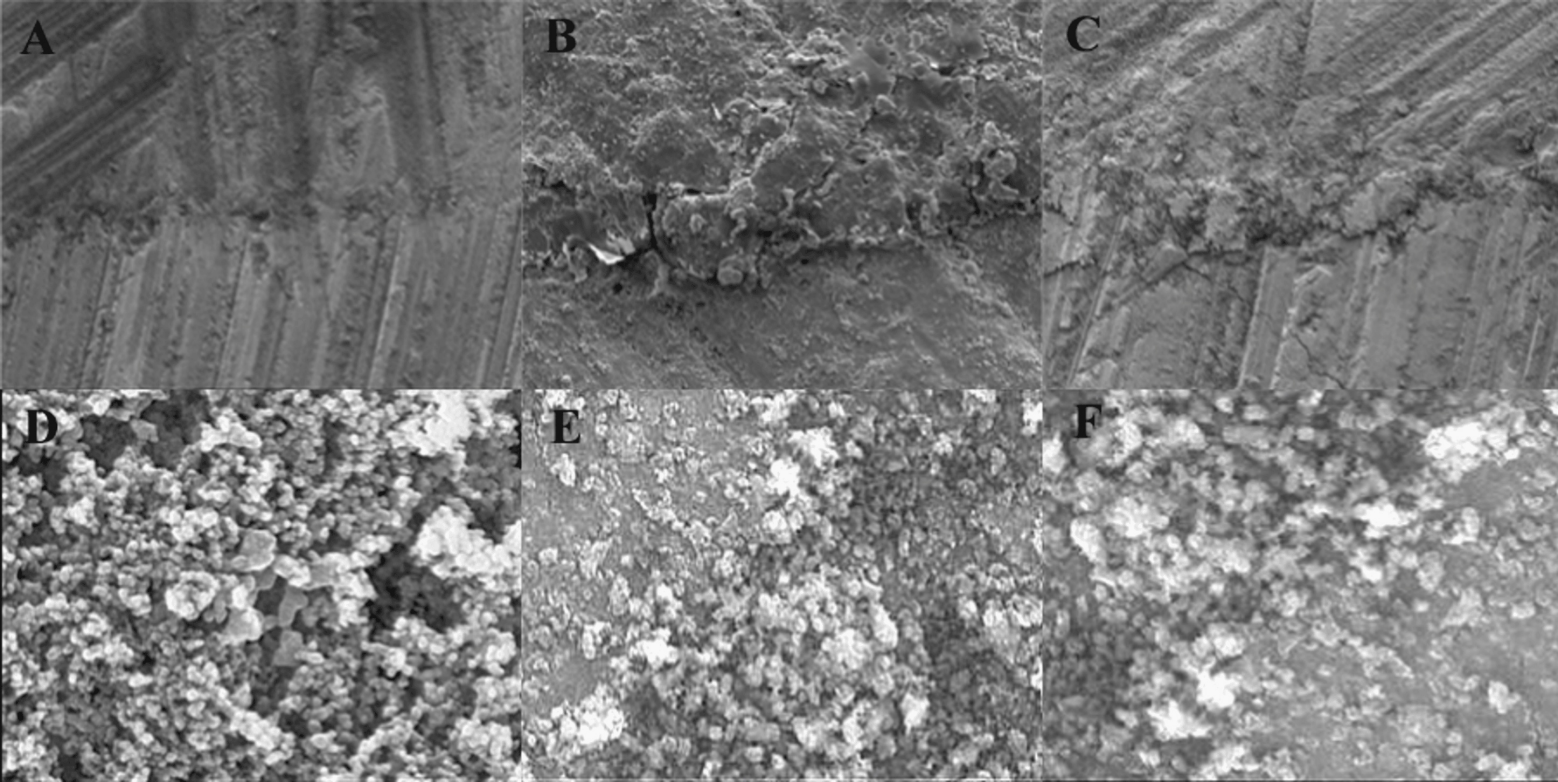
Scanning electron microscopy (SEM) at 2000X magnification showing surface texture. (A) Untreated surface as control group, (B) surface treated with monomer, (C) surface treated with phosphoric acid, (D) surface treated with alumina particles, (E) surface treated with laser combined with phosphoric acid, (F) surface treated with laser and alumina. Original SEM images of samples from the study.

SEM analysis provided critical insights into the microstructural basis of the bond performance. The control surfaces appeared smooth and featureless, correlating predominantly with adhesive failures (Figure 2A). The monomer-treated surfaces exhibited diffuse boundaries with evidence of resin interpenetration, supporting cohesive failure modes (Figure 2B). Phosphoric acid and sandblasting produced distinct topographical changes, irregular pits and abrasive grooves, respectively; however, with limited interfacial blending (Figure 2C, 2D). Laser-irradiated surfaces displayed characteristic molten and recast morphologies, with combinations revealing enhanced microundercuts (Figure 2E, 2F).

## Discussion

The present study investigated the influence of various surface treatments on the tensile strength of heat-cured PMMA denture base resins and heat-polymerized PMMA liners, revealing significant differences among the groups. The superior performance of monomer applications aligns with the principle that chemical modification of the PMMA surface through monomer swelling promotes interdiffusion and chain entanglement at the molecular level. This mechanism facilitates a seamless transition between the existing and newly added resin, creating a robust chemical bond that surpasses mechanical retention. The glossy surface observed during monomer application indicates partial dissolution of the polymer matrix, exposing reactive sites and allowing the liner resin to penetrate and copolymerize effectively [9]. This finding corroborates earlier reports emphasizing the role of monomers in enhancing adhesion by transforming the interface from a distinct boundary to a gradient zone of interpenetrating polymer networks [10,11]. Methyl methacrylate monomers can penetrate deeply into polymeric chains, thereby enhancing the infiltration of adhesive primers [9]. Akin et al. indicated that when soft liners were immersed in isobutyl methacrylate, there was a notable enhancement in their tensile bond strength, resulting in improved performance in all specimens [11].

Phosphoric acid etching, while creating micropores through selective dissolution of the polymer surface, demonstrated a moderate improvement in bond strength. The etched surface likely provided mechanical interlocking sites for the liner resin; however, the limited depth and uniformity of etching may have restricted its efficacy compared with monomer treatment. In contrast to our findings, Gundogdu et al. established that the utilization of 36% phosphoric acid for acid etching of denture bases is the most effective method for increasing bond strength [12]. Almuraikhi T reported the highest mean tensile bond strength with monomers, as compared to surface treatment with phosphoric acid [8]. Similar findings have been reported by Haghi et al. [13].

Sandblasting with alumina particles increased the surface roughness and area; however, its effect was also intermediate, consistent with systematic reviews suggesting that mechanical abrasion alone may not sufficiently activate the chemically inert PMMA surface for optimal bonding with heat-polymerized liners [5]. The inconsistent outcomes of sandblasting in prior literature, where some studies reported enhanced tensile strength with air abrasion [6], may be attributed to variations in particle size, pressure, and duration, underscoring the need for standardized protocols.

The incorporation of Er,Cr:YSGG laser irradiation introduced contemporary dimensions for surface modification. Laser ablation removes the glazed layer and creates micro-retentive craters without introducing foreign particles, potentially preserving the substrate integrity [14]. However, when applied alone or in combination with sandblasting, the results were inferior to those obtained with the monomer. The combination of laser with phosphoric acid, however, has emerged as the most effective hybrid approach, likely due to synergistic effects: laser ablation exposes fresh PMMA chains [15], while subsequent acid etching enhances wettability and microporosity within the ablated craters. This dual mechanism may facilitate deeper resin tag formation and improved interfacial adaptation, as evidenced by the SEM micrographs showing irregular undercut surfaces conducive to mechanical retention. These observations reinforce the hypothesis that bond strength is not solely dependent on roughness but also on the quality of resin infiltration and polymerization across the treated interface [16].

The rejection of the null hypothesis underscores the profound impact of surface preparation on linear adhesion. While all surface treatments improved the tensile bond strength over the untreated control, the hierarchy of efficacy (monomer > laser + phosphoric acid > others) suggests that chemical activation remains paramount for heat-polymerized PMMA systems [9]. This is particularly relevant given that both the substrate and liner undergo identical polymerization cycles, minimizing thermal mismatch but demanding precise surface conditioning to overcome the inherent hydrophobicity and low surface energy of the cured PMMA.

Clinical implications

In clinical practice, monomer application offers a simple, cost-effective, and highly reliable method for ensuring durable linear integration during relining procedures. Their ease of use and consistent outcomes make them suitable for chairside applications, particularly in resource-limited settings. The laser plus phosphoric acid combination, which requires specialized equipment, may be advantageous in cases where maximal mechanical retention is desired without abrasive contamination, such as in patients with resin hypersensitivity. Sandblasting and phosphoric acid alone can serve as adjuncts but should not be relied upon as primary treatments. Clinicians must prioritize treatments that promote chemical bonding to minimize the risk of delamination under cyclic loading, thereby enhancing prosthesis longevity and patient satisfaction.

Limitations

This in vitro study simulated oral conditions; however, it did not replicate the complex intraoral environment involving saliva, dietary solvents, thermal cycling, or dynamic masticatory forces. The use of heat-polymerized PMMA as both the base and liner, while controlling for material variables, may not fully represent clinical reline scenarios involving autopolymerizing or light-cured liners. Specimen geometry and the three-point bending test, although standardized, may not perfectly mimic the clinical stress distribution. Furthermore, the long-term effects of aging beyond 30 days were not evaluated. Future studies should include thermocycling, fatigue testing, and clinical trials to validate these findings.

## Conclusions

This in vitro study confirmed that surface treatments markedly enhanced the tensile strength between the heat-cured PMMA denture base and heat-polymerized PMMA liner. Monomer applications proved to be the most effective, achieving superior chemical bonding via polymer chain interdiffusion. Laser irradiation combined with phosphoric acid has emerged as the optimal hybrid method, synergizing ablation and microporosity for enhanced retention. Individual phosphoric acid and sandblasting offered moderate gains, whereas the laser with sandblasting showed limited synergy. Clinically, a monomer is recommended for reliable, simple relining, and laser-phosphoric acid suits advanced settings. These findings support evidence-based protocols to prevent delamination and improve prosthetic durability.
